# Climbers’ Perception of Hold Surface Properties: Roughness Versus Slip Resistance

**DOI:** 10.3389/fpsyg.2020.00252

**Published:** 2020-03-13

**Authors:** Franz Konstantin Fuss, Yehuda Weizman, Günther Niegl, Adin Ming Tan

**Affiliations:** ^1^Smart Products Engineering Program, Swinburne University of Technology, Melbourne, VIC, Australia; ^2^Climb-on-Marswiese, Sportstättenverein Marswiese, Vienna, Austria

**Keywords:** perception, climbing, handhold surfaces, roughness, slip resistance, grippiness, implicit surface assessment, conscious surface ranking

## Abstract

The more experienced a climber is, the more friction they can impart on a climbing hold surface. The aim of this research was to investigate how the properties of a hold’s surface are perceived and how the perception relates to the amount of friction applied to the hold. The holds’ surface properties are roughness/smoothness and grippiness/slippiness. Fourteen different surfaces with a wide range of property combinations were selected and placed on an instrumented climbing hold, mounted on a bouldering wall, and incorporated into a climbing route. Twenty-two climbers participated in the study. The ratio of friction to normal force (denoted friction coefficient or COF subsequently) was obtained from the sensor data, and the subjective ranking of the surface properties was provided by the participants. The average COF applied to the surfaces ranged from 0.53 (Teflon) to 0.84 (rubber). The surfaces with the lowest and highest grippiness and roughness ranking were Teflon and sandpaper, respectively. The correlation between roughness and COF was insignificant, whereas the correlation of grippiness and COF was significant. This applies to the 22 participants at the group level. At the individual level, 50% (11 climbers) of the participants did not show any correlations between surface properties and COF; eight climbers exhibited correlations between the combined grippiness and roughness (multiple regression) and COF, as well as grippiness and COF; only one climber out of the eight showed an additional correlation between roughness and COF. The results are interpreted in a way that climbers assess a hold’s surface based on grippiness, and not on the roughness, and apply a COF to the hold that reflects the perception of grippiness.

## Introduction

Friction is one of the most important parameters in climbing, as it decides over failure or success when gripping a hold. There is extensive literature on friction in climbing, including a review article by Fuss and Niegl (2012). An additional publication by Fuss and Niegl (2008a) analyzed the friction produced by climbers on a hold instrumented with force transducers during the ladies’ quarterfinal of the 2002 Climbing World Cup in Singapore. Among other parameters, the authors detected that more experienced climbers produce more friction (i.e., a higher friction coefficient) at the hold’s surface. The reasons for this are the following. Firstly, experience is gained through a long-term training effect, which, over time, allows experienced climbers to reduce the margin of error and approach the point of impending slippage. Secondly, experienced climbers exert a smaller force to the handhold compared to less experienced climbers (Fuss and Niegl, 2008a). By shifting the load (normal force) from hands to feet, the coefficient of friction (COF) increases (Fuss and Niegl, 2012). Thirdly, experienced climbers do not fatigue that quickly and thereby maintain the normal force at a magnitude that keeps the COF in the substatic regime. Lastly, long-term climbing, specifically outdoors, leads to thicker and rougher skin, which also contributes to a greater COF. At this point, it has to be mentioned that climbers usually slip more often with their feet rather than with the hands.

In contrast to the extensive literature on climbing and friction, to the best knowledge of the authors, there is no literature source on the perception of surface properties of climbing holds.

Perception is a well-researched area within the discipline of Psychology. “Texture perception” is “the experience of any of a number of surface qualities, for example, roughness, smoothness, … stickiness, slipperiness,…” (Lederman, 1982). Several authors such as Stevens and Harris (1962); Ekman et al. (1965), and Verrillo et al. (1999) correlated the perceived roughness of sandpaper to their grit number and discovered that the regression function follows a power law. Ekman et al. (1965) confirmed this behavior also for the relationship between perceived roughness and the COF. Smith and Scott (1996) extended this research to the correlation between perceived slipperiness and the COF of smooth surfaces. Comparable perception research was applied to the perception of fabrics and textiles. Ramalho et al. (2013) measured the kinetic COF of five fabrics against the skin at loads between 0 and 1 N and a sliding speed 35 ± 10 mm/s and found a correlation with two texture properties, namely “rough/smooth” and “adhesive/slippery.” Chen et al. (2015) and Ding et al. (2018) investigated the roughness ranking of fabrics and their relation to the COF.

When handling objects, test persons perceive slipperiness automatically and implicitly under static conditions by adjusting the grip force automatically such that the object does not slip out of their hands (Johansson and Westling, 1984; Cadoret and Smith, 1996). In contrast to this, Grierson and Carnahan (2006) found that for conscious and accurate perception of slipperiness, movement of the fingers over the surface is required. The latter principle is expected to apply to climbers too. When climbing, they assess the properties of a hold based on its size, shape, surface inclination, and surface properties. This is done by consciously moving the palm over the hold at low force during the “setup” phase of the hand contact (Fuss and Niegl, 2008a). Yet, when loading the hold with greater forces during the “crank phase” (Fuss and Niegl, 2008a), the hand and fingers remain statically on the hold, and the friction force exerted on it is expected to be adjusted implicitly. These expectations are intended to be verified in this paper, supported by appropriate hypotheses.

“Friction” is usually expressed as the ratio of the friction force to the normal force, i.e., the friction force normalized to the normal force and referred to as the COF. At the point of impending slippage, we encounter the static COF, whereas beyond the point of impending slippage, i.e., when sliding over a surface, we deal with the dynamic or kinetic COF. Both cases, even if not desired, occur in climbing when the climber slips off a hold. The ratio of the friction force to the normal force before the point of impending slippage, the common case in climbing, is usually not referred to as the COF; however, for simplicity reasons, the ratio will be denoted as the (*sub*static) COF throughout this paper. The hand or fingers will slip off the hold for two reasons:

(1) If the finger flexing muscles fatigue when clinging to a hold, and weight is shifted from hands to feet, then the normal force on the hands or fingers decreases, which in turn causes the COF to increase, approach the point of impending slippage, and finally exceed this point.
(2) In cases without fatigue, if the static COF is misjudged, the COF applied to the surface exceeds the point of impending slippage.

Performance parameters of climbers are mirrored by difficulty parameters related to a climbing route. This means that when a climber produces a low COF on a hold, then this can be interpreted as a low performance of the climber or as an increased difficulty of gripping the hold (Fuss and Niegl, 2008b; Fuss et al., 2013). The more difficult a hold becomes to deal with and the greater is the danger of slipping off, the more dynamic a move becomes (Fuss and Niegl, 2008b). Climbers use “chalk” (magnesium carbonate) for improving friction on the hold (Fuss et al., 2004). However, chalk can have a negative effect, namely reducing the friction, if a hold is already polluted with chalk or if the surface of a hold is smooth (Fuss et al., 2004; Fuss and Niegl, 2012).

That more experienced climbers exert a higher COF to a hold is an expression of long-term training (Fuss and Niegl, 2008a), i.e., extensive exposure to many different hold surfaces indoors and outdoors. However, it is unclear how climbers assess the surface of a hold and what parameter drives them to produce more friction or less friction on the surface. Potential parameters include the roughness of the surface profile and its slip resistance. Subsequently, these two parameters shall be denoted as roughness/smoothness (rough/smooth) and grippiness/slippiness (grippy/slippy). Based on these two parameters, two cardinal hypotheses can be formulated:

Hypothesis 1: The rougher the surface, the smaller is the chance of slipping off the hold and the more friction the climbers apply to the hold.

Hypothesis 2: The grippier (more slip-resistant) the surface, the smaller is the chance of slipping off the hold and the more friction the climbers apply to the hold.

As the surface properties of a hold are a combination of different degrees of roughness and grippiness, these two parameters must be separated, which can only be done by offering various combinations of high/low roughness and grippiness. As these extreme combinations are not represented by commercially available climbing holds, they can only be provided by using surface materials that are currently not common to sport climbing.

The aim of this paper is to investigate these two hypotheses, insofar as how climbers perceive the surface of a hold and which parameter (rough or grippy) they consciously or implicitly give preference to. This perception study was conducted by offering a range of different surfaces to climbers with a combination of different degrees of grippiness and roughness. We assessed the implicit perception by measuring the COF on the surface and the conscious but subjective perception by ranking the surfaces in terms of grippiness and roughness.

## Materials and Methods

### Rationale of the Method

Perception is a subjective parameter. For experienced climbers, judging the surface of a hold is expected to be an implicit task, which has been trained and perfected over the years of climbing. Nevertheless, the outcome or effect of this task can be measured objectively by instrumenting a hold and calculating the ratio of friction to the normal force, i.e., the COF. Rating and ranking a surface not during climbing is equally a subjective task and reflects the conscious perception of a surface.

The terminologies of surface properties to be used in this paper are as follows:

*Grippiness*: represents a *grippy* surface with high slip resistance;
*Slippiness*: represents a slippery or *slippy* surface with low slip resistance;
*Roughness*: represents a surface with a high amplitude of surface asperities;
*Smoothness*: represents a surface with a very low amplitude of surface asperities or without asperities at all.
COF: the ratio of the friction force to the normal force, which is usually *before* the point of impending slippage (“substatic” COF) in climbing, as *at* the point of impending slippage (static COF), the danger of slipping off (sliding off) a hold is imminent (kinetic COF when slipping).

To statistically separate the two parameters of grippiness and roughness, the climbers must deal with both range and the combination of different degrees of roughness and grippiness, and this is done during climbing for measuring the COF and after climbing for ranking the surfaces. The four combinations thus are:

(a) grippy and rough;
(b) grippy and smooth;
(c) slippy and rough;
(d) slippy and smooth.

It is evident that the two hypotheses formulated in the *Introduction* are inapplicable to combinations (b) and (c). Therefore, the two hypotheses are combined into one, for combinations (a) and (d):

Hypothesis 3: The rougher and grippier (or smoother and slippier) the surface, the lower (or higher, respectively) is the chance of slipping off the hold and the more (or less, respectively) friction the climbers apply to the hold.

Furthermore, if opposing parameters are combined, i.e., combinations (b) (smoother and grippier) and (c) (rougher and slippier), then the options for climbers are putting more importance on either:

– Grippiness (such that the friction increases as grippiness does); or
– Roughness (such that the friction increases as roughness does); or on
– The average of grippiness and roughness, resulting in average friction.

To confirm Hypothesis 3 and assess the additional three options, the following investigations were carried out based on the processed data of the instrumented hold (with 14 different surfaces) and the subjective ranking of roughness and grippiness:

– Hold surfaces and their difference in the COF, roughness, and grippiness;
– Four combinations of roughness and grippiness in terms of which combination drives the climber to produce the highest and the lowest COF; and
– Difference between climbers in terms of the individual and the combined influence of roughness and grippiness on their COF produced on each hold.

These investigations come down to answering the following three questions.

– Does “the property” of the hold’s surface influence the COF produced by a climber in a sense that perception of “the property” triggers the amount of friction applied “automatically” or implicitly to the hold?
– Which parameter are the climbers implicitly going for when assessing or gripping a hold’s surface during climbing: roughness/smoothness or grippiness/slippiness or both?
– Are individual climbers really “implicitly aware” of what they are doing on a hold, e.g., because of a long-term training effect or intuitively?

### Surface Materials for the Instrumented Hold

The rationale for the selection of materials was driven by the intention to have several surfaces for all the four combinations explained above. Naturally, combinations (b) and (c) are difficult to achieve.

The theoretical starting point for finding surfaces that fit into all four combinations would have been by using commercially available artificial climbing holds of different brands. This would have been unfeasible for various reasons.

– To minimize the variables of this study and for comparative reasons, the surfaces had to be of the same size and shape, preferably flat.
– Flat (plane) surfaces also reduce the complexity of the instrumentation, as for a curved surface, we need two transducers (surface curved only in one direction) instead of one. In a curved surface, the average COF is determined at (and tangent to) the COP (center of pressure), which can only be calculated when measuring the moment from two transducers.
– It is extremely difficult to find holds that have an almost flat surface segment and this from several brands.
– Purchasing different holds and cutting them to size, i.e., to the flat segment only (for mounting them on the transducer), does not guarantee that we obtain the required combinations and ranges of grippiness and roughness for drawing convincing conclusions.

Consequently, some materials were selected from the stock we had at our Health and Sports Technologies Laboratory, and others were purchased at a hardware supermarket. The selection criteria were twofold, namely, (1) find the required range of roughness and grippiness combinations, and (2) introduce materials that are common (rocks, sand) and uncommon (e.g., rubber, carpet) in climbing.

The justification of the first selection criterion is provided by the range and combinations of grippiness and roughness, specifically by unusual combinations such as combinations (b: grippy and smooth) and (c: slippy and rough). These special combinations separate the two properties and allow for determining whether the climbers assess a surface based on grippiness or roughness.

The justification of the second selection criterion is provided by the fact that not all special and extreme combinations can be obtained from surfaces common in climbing. For example, the combination of grippy and smooth is typical for rubber but atypical in a rockface. To prevent climbers from associating one extreme surface property with a common material (e.g., rough with a rock surface) and another property with an uncommon material (e.g., smooth with a polymeric surface) and thereby recognize a pattern that could influence their decisions or reactions, we had to provide a variety of common and uncommon surfaces. We could have used smooth stone surfaces instead of polymeric ones, but they would not have been that grippy as rubber is.

**FIGURE 1:**
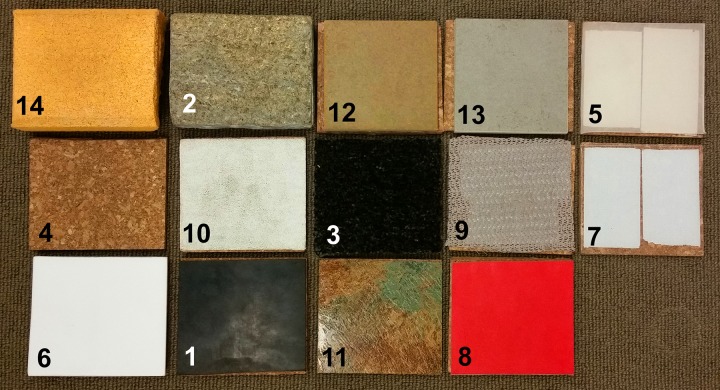
Fourteen hold surfaces numbered **1**–**14** (according to Table 1)—(1) black rubber; (2) mica schist; (3) carpet; (4) cork; (5) silicone rubber; (6) Teflon; (7) translucent plastic; (8) leather; (9) magic stop; (10) sandpaper; (11) green tile; (12) ceramic tile brown; (13) ceramic tile gray; (14) sandstone; surfaces no. 5 and 7 are translucent and therefore the double-sided adhesive tape, used for attaching the surfaces to a cork carrier, is visible.

During the selection process, different surface materials were assessed manually, and finally, 14 surfaces were selected (Figure 1 and Table 1).

**TABLE 1:** Description of the 14 different surfaces.

Surface ID number	Name	Description
1	Black rubber	A mixture of natural and synthetic rubber; smooth surface; color: black
2	Mica schist	Rock based on mica (phyllosilicates) and quartz arranged in layers; color: grayish/bluish/greenish
3	Carpet	Polypropylene carpet, 5-mm tuft length; color: gray
4	Cork	Cork tile, 6-mm thickness, color: brown
5	Silicone rubber	Smooth surface; color: whitish translucent
6	Teflon	PTFE (polytetrafluoroethylene); smooth surface; color: white
7	Translucent plastic	Clear Vinyl sheet, 0.75-mm thickness; smooth surface; color: translucent
8	Leather	Tanned cowhide used for producing Australian-Rules (AFL) footballs; color: red
9	Magic stop	Non-slip rubber used as slip protector underneath carpets, color: gray
10	Sandpaper	Sandpaper, 80 grit, color: white
11	Green tile	Vinyl floor tile “green slate”; color: brown/green
12	Ceramic tile brown	Ceramic tile, color: brown
13	Ceramic tile gray	Ceramic tile, color: gray
14	Sandstone	Brick, color: yellowish

### Instrumented Hold

**FIGURE 2:**
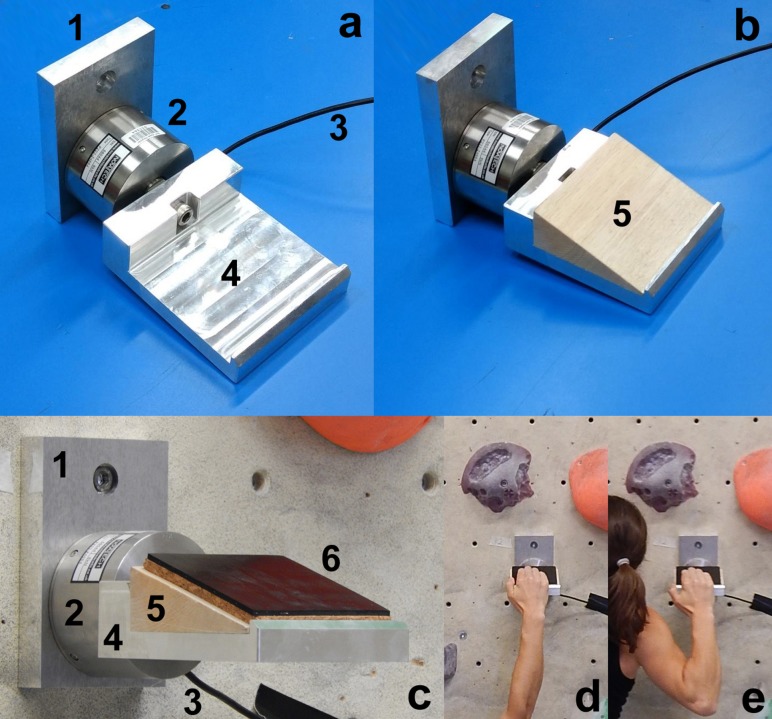
Instrumented hold; (**a**) hold—transducer assembly; 1 = plate (for mounting the assembly on the climbing wall); 2 = force transducer; 3 = transducer cable; 4 = aluminum frame of the hold; **(b)** assembly with a 20° wooden wedge (5); **(c)** assembly mounted on the wall with black rubber surface (6); **(d,e)** position of the climber’s hand on the hold.

A hold was designed in Solidworks 2019 (Dassault Systèmes, Nashville, TN, United States) and manufactured of aluminum (Figure 2) that allows for inclining the surface at three different angles (0, 9, and 20°) and for quickly changing the surface materials. The size of the surfaces was 100 mm × 100 mm so that the skin surface distal of the fingers’ MCP joints (metacarpophalangeal joints) fit entirely on the hold. The design of the hold eliminated the size, shape, and inclination factor and confined the variability to the surface properties. The hold was connected to a 3 DOF strain-gauged force transducer (5 kN in each direction; type F233, Novatech Measurements, Ltd., East Sussex, United Kingdom), which in turn was mounted on an aluminum plate (to be attached to a climbing wall). The force transducer was connected to a microcontroller (TEENSY 3.1, 32-bit ARM Cortex-M4 72 MHz CPU, PJRC, Sherwood, OR, United States), and the data were recorded at a sampling rate of 10 Hz. The accuracy of the force transducer was verified after assembly with various weights (10–200 N), introduced at different locations within the placement area of the 14 different surfaces.

### Climbing Route

**FIGURE 3:**
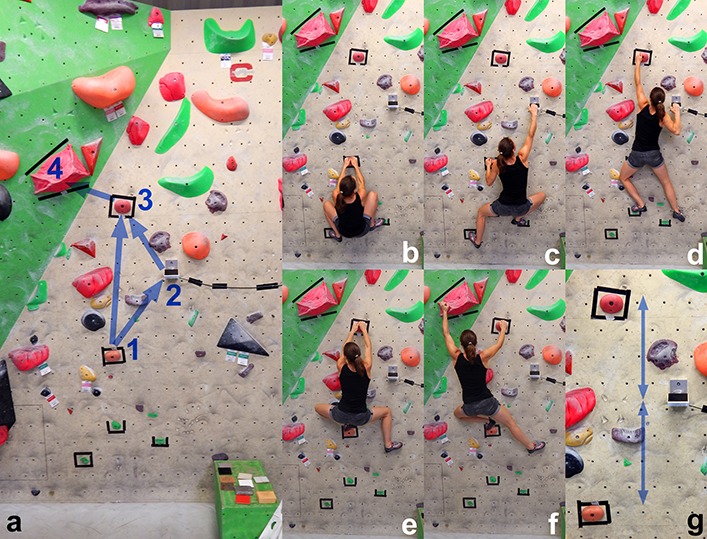
Climbing route and moves; **(a)** bouldering wall with mounted instrumented hold (2; cable to the right of the hold); the blue arrows indicate the movement sequence between the handholds (1, 2, 3, 4); some of the surfaces of the instrumented hold are visible in the lower right corner; (**b–f)** movement sequences; **(g)** enlarged handhold area; the distances indicated by the two blue arrows are 0.605 m each.

The climbing route was designed by one of the participants together with the authors of this paper on an indoor bouldering wall. It consisted of four moves (Figure 3): both hands on the starting hold (hold no. 1, jug); right hand to the instrumented hold (hold no. 2); left hand to hold no. 3 (jug); right hand also to hold no. 3; and finally left hand to hold no. 4 (large hold) after which the climbers jumped off the wall. The vertical distances between hold nos. 1, 2, and 3 were 0.605 m each.

The inclination of the hold’s surfaces was −20° (sloping downward) with respect to the coordinate system of the force transducer. The hold was mounted on a −9° inclined (overhanging) wall so that the inclination angle of the hold with respect to the horizontal axis of the global coordinate system was −29°. This total inclination angle of −29° requires a minimum COF of 0.5543 (tan 29°) for preventing slipping off the hold. The total inclination angle was selected such that there is a realistic chance of slipping off a surface, at least off the most slippery one (surface no. 6, Teflon).

Note that if the minimum COF is smaller than the actual static COF, then the minimum COF is substatic, and the climber will not slip off the hold. If the minimum COF (required for a specific inclination angle) is greater than the actual static COF, then the minimum COF is either dynamic on velocity-strengthening (the COF increases as the sliding velocity does; Fuss, 2012), resulting in slipping (if the increased dynamic COF does not exceed the minimum COF) or slip-stick (if the increased dynamic COF exceeds the minimum COF and subsequently oscillates between static and dynamic), or can never be reached on velocity-weakening (the COF decreases as the sliding velocity increases; Fuss, 2012), resulting in slipping off the hold. The slip-stick phenomenon will hardly happen as the surface of climbing holds is usually curved and therefore the minimum COF increases when slipping off a hold.

The participants had to grip the surface of the instrumented hold with the open hand grip (Figures 2d,e, 3c).

### Participants

Twenty-two climbers participated in this study: 8 female and 14 male climbers; 1 left-hander and 21 right-handers. Their age was 27.27 ± 8.47 years; climbing experience 11.34 ± 7.32 years; redpoint grading (lead climbing) 21.11 ± 4.17 (IRCRA Climbing Grades; Draper et al., 2015) corresponding to an average of 7c French grading; onsight grading (lead climbing) 17.84 ± 4.24 (IRCRA Climbing Grades; Draper et al., 2015) corresponding to an average of 7a+ French grading (for conversion to other grading systems, see Draper et al., 2015); body height 1.74 ± 0.073 m; body mass 67.5 ± 9.9 kg; BMI 22.12 ± 2.16 kg/m^2^.

This study was granted ethics approval by the Swinburne University Human Ethics Committee (approval no. 20191290-1680) and adhered to the Declaration of Helsinki.

### Experimental Procedure

The participants filled in an informed consent form before the start of the experimental procedure, which includes their anthropometric data and climbing performance. The consent form informed the climbers of the instrumented hold and that force data applied to the hold were recorded.

The purpose of the study was not revealed to the participants before climbing. Any information on surface properties (grippiness, roughness) could influence the participants by paying more attention to the surface than usual and thereby distract them from unbiased climbing.

The climbers were not allowed to use “chalk” while climbing. There are three reasons for this. First, using chalk on smooth polymeric surfaces considerably decreases the COF. In a study by Fuss et al. (2004), the average static COF between Perspex and a dry hand was 1.475, whereas between Perspex and the hand covered with powder chalk or dried liquid chalk, it reduced the average static COF to 0.722 and 0.634, respectively. Such a reduction by 50% explains that when using chalk, the effect of the grip-enhancing agent is assessed rather than the surface properties. We had three polymeric surfaces and three elastomeric surfaces among our surface samples. Secondly, the surfaces should not get polluted and thereby should not change their properties over time. This could have been prevented by cleaning, i.e., washing the surfaces after each climb. However, some surfaces change their properties when being in contact with water. This applies to the cork and carpet surfaces, which should have been dried completely after washing, and to the sandpaper that tends to disintegrate on contact with water. Alternatively, the surfaces could have been replaced throughout the experiments, which would have resulted in a too high workload. Thirdly, the participants had to rank the grippiness (and the roughness) of the surfaces after the climb, which again, when using chalk, would have resulted in assessing the effect of the grip-enhancing agent, which defied the purpose of the study.

A clean towel was provided for the participants for cleaning their hands before climbing in the case of having sweaty hands or hands covered with residues of chalk.

The climbers were informed that, depending on how difficult it is to have a firm grip on the surfaces, both static and dynamic moves are allowed.

Before starting the experiment, the climbers tested the route a couple of times with a specific hold to avoid a learning effect during climbing, which could influence the results. Before starting the experiment, the force transducer was switched on for recording of the data. Before each of the 14 climbs, a new surface was placed on the instrumented hold in a random order, and the surface ID no. was recorded on the consent form. The random order of the different surfaces was required such that the property of a preceding surface influencing the perception of the following one does not produce a systematic error. Any comments expressed by the participants during climbing were recorded by noting them down on the consent forms. After completing the 14 ascents, the data recording was stopped, and the participants were informed of the principles of roughness/smoothness and grippiness/slippiness (slip resistance). Subsequently, they were asked to rank the 14 surfaces with respect to grippiness first, followed by roughness. For this purpose, the 14 surfaces were placed on a wooden box at the bottom of the climbing route; the climbers slid their fingers over the 14 surfaces and lined up the surfaces from the lowest to greatest grippiness and the lowest to greatest roughness. The sequences of the surfaces were recorded on the consent form after each ranking exercise (grippiness from 1 to 14; roughness from 0 to 10, with 0 assigned to the perfectly smooth surfaces). Finally, the climbers were asked to indicate whether they assess a hold for roughness or grippiness (slip resistance) when climbing. Any further feedback arising from the last question was recorded too.

### Data Processing

Our software provided the data as vertical and horizontal forces (in Newtons) applied to the force transducer. After offset correction (the surfaces placed on the hold had different masses), the forces were rotated by 20° (inclination of the hold’s surface with respect to the coordinate system of the force transducer) and thereby converted to normal forces (perpendicular to the surface) and friction forces (parallel to the surface). For each loading period related to a specific surface, the following data were extracted: maximum friction and normal forces, the COF at the maximum normal force, and average friction and normal forces (*F* and *N*, respectively). The average COF was calculated as a weighted average COF (weighted with respect to the normal force *N*), as the resolution and measurement errors of the force transducer at small forces can produce an excessive COF and therefore an incorrect (unweighted) average COF. The weighted average COF results from

(1) $$C\,O\,F_{w\,e\,i\,g\,h\,t\,e\,d}=\frac{\sum \left(C\,O\,F\cdot N\right)}{\sum N}=\frac{\sum F}{\sum N}=\frac{\bar{F}}{\bar{N}}$$

considering that COF = *F*/*N* and that the loading periods of *F* and *N* were equal.

### Statistical Analysis

For the surface analysis, the averages and standard deviations of the weighted average COF, grippiness ranking, and roughness ranking were calculated. The averages served for comparison and further ranking, whereas the standard deviations informed of the parameter consistency across the different surfaces.

For hypothesis testing, the combinations of grippy, slippy, rough, and smooth were compared with unpaired *t*-tests and ANOVA. The normal distribution of the data was verified with the Shapiro–Wilk test. For the *t*-tests, the variances were assessed with the *F*-test, and the significance of the combinations in the ANOVA test was assessed with the following *post hoc* tests: Tukey, Scheffe, Bonferroni, and Holm. For significance testing, α was set to 0.05.

For the climber analysis, multiple and single regressions were analyzed: grippiness + roughness vs. COF, grippiness vs. COF, and roughness vs. COF. This served for quantifying the influence of the surface ranking on the COF, e.g., if the *R*^2^ value of grippiness vs. COF was 0.4, then 40% of the magnitude of the COF can be explained from the degree of grippiness. The conditions imposed on the regressions was that all trends had to be positive and significant (α = 0.1), positive because the COF is expected to increase as grippiness and roughness do. From the three *R*^2^ values of multiple and single regressions, the combined influence was calculated from the sum of the *R*^2^ of the single regressions minus the *R*^2^ of the multiple regression. The individual influences (semipartial correlations) of grippiness and roughness were calculated from the single regression *R*^2^ minus the combined influence. The influences were expressed as a percentage, resulting from 100 ^∗^ *R*^2^. The condition imposed on the combined influence was that it had to be positive. Negative combined influence indicates that there is no combined influence.

## Results

### Surface Analysis

The surface properties are listed in Table 2. The highest COF was produced on the black rubber surface (0.841 on average) followed by sandpaper (0.823); the lowest one was on Teflon (0.529 on average), followed by mica schist (0.634). Note that black rubber and Teflon surfaces were perfectly smooth. The surface that was ranked the highest for slip resistance was sandpaper followed by black rubber; the lowest was Teflon followed by translucent plastic. The surface that was ranked the highest for roughness was sandpaper followed by mica schist; the lowest was, evidently, the four perfectly smooth surfaces. Interestingly, although black rubber ranked higher than sandpaper while climbing, the climbers considered the rough sandpaper grippier than the smooth black rubber surface.

**TABLE 2:** Surface properties; highest rank = best performing, i.e., greatest COF, grippiest, roughest, 100% success (not slipping off any surface); note that the inclination angle of the hold’s surface (−29°) requires a minimum COF of 0.5543, and that the average COF of Teflon was below this threshold; note that an average ± standard deviation of 0 ± 0 indicates a perfectly smooth surface.

Surface ID no.	Name	Weighted average COF avg ± std	Rank	Grippiness ranking avg ± std	Rank	Roughness ranking avg ± std	Rank	Success rate (%)	Rank
1	Black rubber	0.841 ± 0.125	14	10.545 ± 3.555	13	0 ± 0	0	100	7
2	Mica schist	0.634 ± 0.092	2	7.045 ± 3.000	5	8.318 ± 1.729	9	90.9	5
3	Carpet	0.706 ± 0.094	8	8.000 ± 3.309	6	5.318 ± 2.533	5	81.8	3
4	Cork	0.760 ± 0.093	10	8.045 ± 2.081	7	3.636 ± 1.293	3	100	7
5	Silicone rubber	0.687 ± 0.116	4	4.727 ± 3.355	4	0 ± 0	0	77.3	2
6	Teflon	0.529 ± 0.090	1	1.500 ± 0.913	1	0 ± 0	0	40.9	1
7	Translucent plastic	0.718 ± 0.107	9	3.136 ± 2.965	2	0 ± 0	0	86.4	4
8	Leather	0.766 ± 0.106	11	8.182 ± 2.702	8	2.091 ± 1.477	1	100	7
9	Magic stop	0.788 ± 0.121	12	9.500 ± 2.483	12	4.818 ± 2.062	4	95.5	6
10	Sandpaper	0.823 ± 0.119	13	13.409 ± 1.141	14	9.273 ± 1.486	10	100	7
11	Green tile	0.692 ± 0.121	5	4.545 ± 2.614	3	2.591 ± 1.894	2	90.9	5
12	Ceramic tile brown	0.706 ± 0.096	7	8.477 ± 2.872	9	6.477 ± 2.073	8	81.8	3
13	Ceramic tile gray	0.699 ± 0.110	6	8.977 ± 3.041	10	6.432 ± 1.978	7	95.5	6
14	Sandstone	0.680 ± 0.078	3	9.000 ± 2.911	11	6.318 ± 1.729	6	100	7

In terms of the standard deviations, for the weighted average COF, the least controversial surfaces (with the smallest standard deviation) were sandstone, Teflon, and mica schist (probably because climbers are more familiar with rocky surfaces and because Teflon was both smooth and most slippy); the most controversial were black rubber and the green tile (black rubber probably because it was smooth and the most grippy surface).

In terms of slip resistance ranking, Teflon and sandpaper were the least controversial ones (most consistent ranking); black rubber and silicone rubber were the most controversial ones (most inconsistent ranking).

**FIGURE 4:**
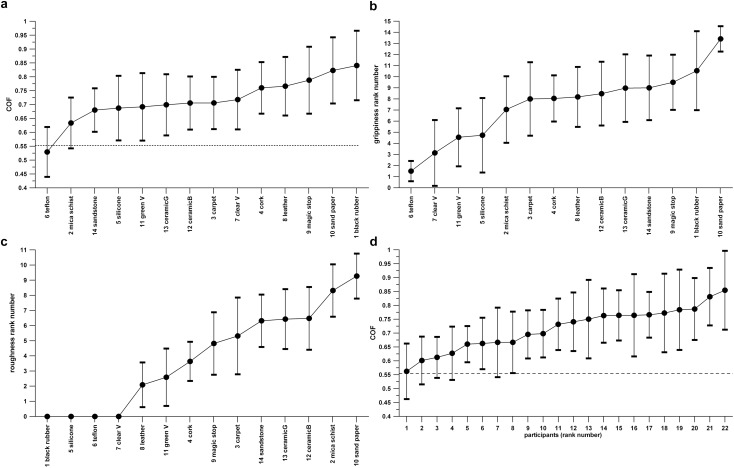
Fourteen hold surfaces and their average (± standard deviation) coefficient of friction (**a**), grippiness ranking (**b**), and roughness ranking (**c**); and the climbers’ COF (average ± standard deviation); (**d**); lowest to highest from left to right; the dashed line in subpanels (**a**,**d**) indicates the minimum COF of 0.5543 required for holding a surface of an inclination angle of –29°.

Figure 4 shows the distribution of average ± 1 standard deviation of COF (Figure 4a), slip resistance ranking (Figure 4b), and roughness ranking (Figure 4c).

In terms of roughness ranking, the least controversial surfaces were leather and cork, and the most controversial were carpet and the brown ceramic tile.

Five out of 14 surfaces were held by all climbers (success rate of 100%). The two surfaces with the least success percentage were silicone rubber (surface no. 5) with 77.3% and Teflon (surface no. 6) with a 40.9% success rate.

It seems that the success rate can be explained from the average COF and the grippiness rather than from the roughness. Correlating the success rate (%) against the average COF, average grippiness rank, and the average roughness rank returns *R*-values of +0.6187 (*p* = 0.0008), +0.5292 (*p* = 0.0032), and +0.1564 (*p* = 0.1620), respectively. Correlating the ranked success rate against the ranked average COF, ranked average grippiness rank, and the ranked average roughness rank returns *R*-values of +0.3316 (*p* = 0.0312), +0.5024 (*p* = 0.0045), and +0.0661 (*p* = 0.3747), respectively. These data confirm that the positive regression trends of the success rate are significant only for an average COF and average grippiness. Whether this result suggests that the COF depends more on the grippiness rather than on the roughness will be examined subsequently.

Figure 4d shows the distribution of average ± 1 standard deviation of the individual climbers’ COF. The weighted average COF ranges considerably over 0.292, from 0.562 to 0.855. The smallest average COF is just a little over the minimum COF required for holding the inclined surface of the instrumented hold.

The individual climber’s COF (average across all 14 holds of the weighted average COF per hold) correlated significantly with the climbing experience (in years) through a positive trend (*R*^2^ = 0.2000, i.e., 20% of the COF were explained from the climbing experience; *p* = 0.0362; α = 0.1). The same trend was seen when correlating all weighted COF data of each hold and the climber with the climbing experience (*R*^2^ = 0.0684; *p* < 0.0001, α = 0.1). The correlations of the individual climber’s COF or all weighted COF data with RP or OS were non-significant.

### Hypotheses Testing

**FIGURE 5:**
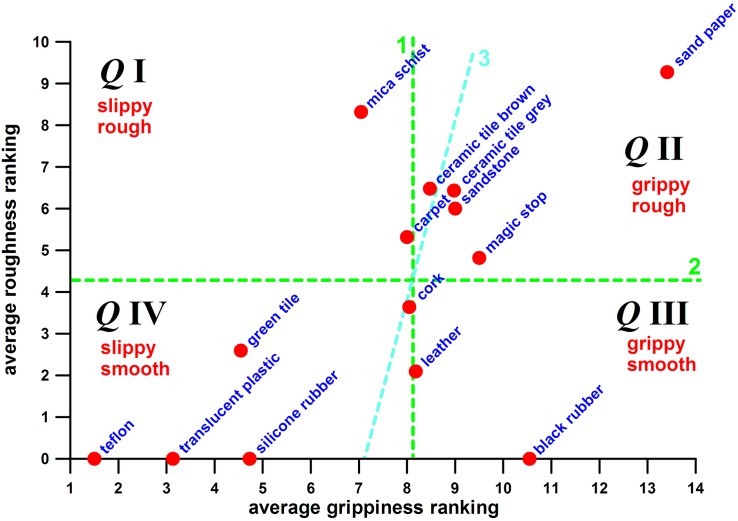
Hold surface property map, average roughness ranking vs. grippiness ranking; (*Q*I–*Q*IV) Quarters I to IV; (1) dashed line dividing the surfaces into a slippy and grippy half; (2) dashed line dividing the surfaces into a rough and smooth half; (3) tilted line 1 required for a more even distribution of the surfaces across the four quarters.

Figure 5 shows the distribution of the two parameters, average grippiness ranking and average roughness ranking. Dividing both parameters into two halves of equal number of data isolates the four combinations of grippiness/slippiness and roughness/smoothness and divides Figure 4 into four quarters.

The extreme representatives of each quarter were:

– Quarter I (slippy and rough): mica schist;
– Quarter II (grippy and rough): sandpaper;
– Quarter III (grippy and smooth): black rubber;
– Quarter IV: (slippy and smooth): Teflon.

As already mentioned earlier, the two combinations of opposing properties—grippy and smooth, and slippy and rough—are difficult to achieve and therefore underrepresented in the graph. The number of surfaces in each quarter is 2, 5, 2, and 5 from Quarter I to Quarter IV (Figure 5). To obtain a more even number distribution (3, 4, 3, 4), one data point in each of the quarters with five data is moved to the quarters with two data. The two data points are ceramic tile brown (moved from Quarter II to I) and cork (moved from Quarter IV to III). This data inclusion is justified as both surfaces have equal grippiness ranking and weighted average COF (*p* = 0.5710 and *p* = 0.0617, respectively; two-tailed unpaired *t*-test for normally distributed data sets). The more even data point distribution across the four quarters avoids that a single high- or low-performing surface could dominate one quarter.

To test Hypothesis 3, Quarters II and IV are compared first (grippy + rough vs. slippy + smooth), as to the weighted average COF:

– Mean COF grippy + rough: 0.7477
– Mean COF slippy + smooth: 0.6567

*p*-value: 3.99 × 10^–6^ (two-tailed unpaired *t*-test, equal variances, normal data distribution); effect size *d* = 0.7069 (medium effect).

Hypothesis 3 is thereby confirmed, namely that the surface property combination “grippy + rough” has a significantly greater COF than the combination “slippy + smooth.”

Which one of the two properties (grippy/slippy or rough/smooth) influences the most the difference between the two averages? This is tested by comparing two halves of the diagram in Figure 5, namely grippy vs. slippy; and rough vs. smooth:

(1) Grippy vs. slippy
  – Mean COF grippy: 0.7655
  – Mean COF slippy: 0.6674
  – *p*-value: 4.21 × 10^–12^ (two-tailed unpaired *t*-test, equal variances, normal data distribution); effect size *d* = 0.7616 (medium effect).
(2) Rough vs. smooth
  – Mean COF rough: 0.7194
  – Mean COF smooth: 0.7135
  – *p*-value: 0.6851 (two-tailed unpaired *t*-test, *un*equal variances, normal data distribution); effect size *d* = 0.0463 (very small effect).

Grippy surfaces have a significantly higher mean COF than slippy ones. Although the mean COF of smooth surfaces is slightly smaller than the one of rough surfaces (expectedly), the two mean COFs are not significantly different, with a very small effect size. These results indicate that climbers give preference entirely to grippiness (slip resistance) rather than to roughness.

To verify the group differences, the weighted average COF data of each quarter are compared to each other with an ANOVA test, with the following outcome:

– *p*-value of the ANOVA test: 1.05 × 10^–11^

*p*-values of the *post hoc* tests plus their interpretations:

– Grippy + rough vs. slippy + rough: *p* ≤ 0.009 (significant difference; grippy > slippy, rough = rough, difference comes from grippy/slippy); effect size: *d* = 0.5122 (moderate effect).
– Grippy + rough vs. grippy + smooth: *p* ≥ 0.064 (equal averages; grippy = grippy, rough = smooth [because of *p* > 0.05]); effect size: *d* = 0.3222 (small effect).
– Grippy + rough vs. slippy + smooth: *p* ≤ 0.001 (significant difference; grippy > slippy, rough = smooth, difference comes from grippy/slippy); effect size: *d* = 0.7069 (moderate effect).
– Slippy + rough vs. grippy + smooth: *p* ≤ 0.001 (significant difference; grippy > slippy, rough = smooth, difference comes from grippy/slippy); effect size: *d* = 0.8344 (large effect).
– Slippy + rough vs. slippy + smooth: *p* ≥ 0.194 (equal averages; slippy = slippy, rough = smooth); effect size: *d* = 0.1947 (very small effect).
– Grippy + smooth vs. slippy + smooth: *p* ≤ 0.001 (significant difference; grippy > slippy, smooth = smooth, difference comes from grippy/slippy); effect size: *d* = 1.0291 (large effect).

These results confirm the outcome of the initial *t*-tests.

The difference between the mean COFs of grippy + rough and grippy + smooth is not significant (small effect size); the same applies to slippy + rough vs. slippy + smooth (very small effect size).

What these two combinations have in common are the properties of “grippy” and “slippy,” respectively, which proves that the non-significant difference and the small effect size must come from “rough” and “smooth.”

The opposite is true for the combinations grippy + rough vs. slippy + rough and grippy + smooth vs. slippy + smooth, with significant differences and moderate to large effect sizes. What these two combinations have in common are the properties of “rough” and “smooth,” respectively, which proves that the significant difference and moderate to large effect sizes must come from the difference between “grippy” and “slippy.”

The results of the ANOVA analysis, applicable to all combinations, are that there is a significant difference between the surface properties of “grippy” and “slippy” but not between rough and smooth.

### Climber Analysis

The individual climbers are analyzed as to their surface property preference (grippiness/slippiness or roughness/smoothness) with individual and multiple regression analyses (both properties vs. weighted average COF and each property individually vs. COF). The results to be compared are the trends of the individual regression analysis (positive trends for both regressions), the coefficients of determination (R^2^) of individual and multiple regressions, the individual (semipartial correlations) and combined influences of both properties on the COF, and the amount of the COF not explained from both properties (grippiness/slippiness and roughness/smoothness). Table 3 shows the correlation data of each participant as well as their classification type (1–5).

**TABLE 3:** Influence of the holds’ surface properties on the COF (coefficient of friction).

Participant no.	100 * *R*^2^ of multiple regression	100 * *R*^2^ of grippy/slippy vs. COF	100 * *R*^2^ of rough/smooth vs. COF	Combined influence on COF (%)	Individual influence of grippy/slippy on COF (%)	Individual influence of rough/smooth on COF (%)	Unexplained influence (%)	Classification type
All participants, all data	**18.63**	**16.74**	n/a	n/a	n/a	n/a	**81.37**	**3**
All participants, average data of each hold	**80.11**	**56.96**	n/a	n/a	n/a	n/a	**19.89**	**3**
4	n/a	n/a	n/a	n/a	n/a	n/a	**100**	**1**
5	n/a	n/a	n/a	n/a	n/a	n/a	**100**	**1**
7	n/a	n/a	n/a	n/a	n/a	n/a	**100**	**1**
11	n/a	n/a	n/a	n/a	n/a	n/a	**100**	**1**
13	n/a	n/a	n/a	n/a	n/a	n/a	**100**	**1**
14	n/a	n/a	n/a	n/a	n/a	n/a	**100**	**1**
15	n/a	n/a	n/a	n/a	n/a	n/a	**100**	**1**
16	n/a	n/a	n/a	n/a	n/a	n/a	**100**	**1**
17	n/a	n/a	n/a	n/a	n/a	n/a	**100**	**1**
18	n/a	n/a	n/a	n/a	n/a	n/a	**100**	**1**
20	n/a	n/a	n/a	n/a	n/a	n/a	**100**	**1**
8	n/a	**58.35**	n/a	n/a	n/a	n/a	**41.65**	**2**
12	n/a	**30.16**	n/a	n/a	n/a	n/a	**69.84**	**2**
19	n/a	**20.97**	n/a	n/a	n/a	n/a	**79.03**	**2**
1	**47.48**	**40.99**	n/a	n/a	n/a	n/a	**52.52**	**3**
10	**51.51**	**45.18**	n/a	n/a	n/a	n/a	**48.49**	**3**
21	**59.69**	**57.57**	n/a	n/a	n/a	n/a	**40.31**	**3**
22	**67.35**	**56.34**	n/a	n/a	n/a	n/a	**32.65**	**3**
2	**36.82**	**36.81**	n/a	**7.96**	**28.85**	n/a	**63.18**	**4**
3	**46.85**	**44.13**	n/a	**7.2**	**36.93**	n/a	**53.15**	**4**
6	**43.98**	**43.86**	n/a	**18.92**	**24.94**	n/a	**56.02**	**4**
9	**54.96**	**43.12**	**42.83**	**30.98**	**12.13**	**11.84**	**45.04**	**5**
Type 1, all participants, all data	n/a	**7.66**	n/a	n/a	n/a	n/a	**92.34**	**2**
Types 2–5, all participants, all data	**29.45**	**29.00**	**2.60**	**2.15**	**26.85**	**0.45**	**70.55**	**5**
Type 1, all participants, average data of each hold	n/a	**33.44**	n/a	n/a	n/a	n/a	**66.56**	**2**
Types 2–5, all participants, average data of each hold	**72.31**	**66.95**	n/a	**1.58**	**65.37**	n/a	**27.69**	**4**

*Bold values indicate percentages and classification types.*

Type 1, in 11 out of 22 participants (50%), is characterized by insignificant trends in all regressions (multiple and single individual ones). The unexplained influence was therefore set to 100%.

Type 2 (13.64%) shows a significant correlation between grippy/slippy and COF but an insignificant correlation between rough/smooth and COF, which is, moreover, negative. Therefore, the multiple regression was not calculated as the roughness-related coefficient of the multiple regression equation had a negative sign, which turns the originally negative correlation into a positive one. The unexplained influence was determined from the *R*^2^ of the correlation between grippy/slippy and COF.

Type 3 (18.18%) shows a significant correlation between grippy/slippy and COF but an insignificant correlation between rough/smooth and the COF, which is positive. Therefore, multiple regression was calculated. The combined and individual influences were not determined because the combined influence was negative.

Type 3 was also found when using the data of all participants combined (with low *R*^2^ values), as well as the average data of each hold across all participants (with high *R*^2^ values; Table 3).

Type 4 (13.64%) is comparable to Type 3 with the difference that the combined influence was positive; this allowed identifying the individual influence of the grippiness/smoothness on the COF.

Type 5 (4.55%) was represented by only one participant, exhibiting significant multiple and single individual regressions and combined and individual influences on the COF. The multiple regression *R*^2^ was 55% (i.e., 55% of the COF could be explained from combined grippiness and roughness), and the single individual regression *R*^2^ was 43% each. This led to a 31% combined influence and 12% individual influences each of the two properties on the COF. This was the only participant that showed a significant influence of the roughness on the COF and this at the same level as the grippiness.

In types 2–5, 21–58% (43.40 ± 11.50%) of the magnitude of the COF could be explained from grippiness. Grouping the data of all participants of types 2–5 together, then 29% of the COF could be explained from grippiness; taking the average data of each hold across all participants, then 67% of the COF could be explained from grippiness.

In types 1–4, the roughness did not have any influence on the COF; neither did the grippiness in type 1, i.e., in 50% of the participants.

Surprisingly, grouping the data of all participants of types 2–5 together, the group performance corresponded to type 5. However, the percentage influences of roughness on the COF, the combined influence of grippiness and roughness, and the exclusive influence of roughness were very small (≤2.6%) but nevertheless significant. This stands in contrast to the average data of each hold across all participants, where the influence of roughness was insignificant, resulting in type 4. Three times the number of average data [i.e., 42 surfaces instead of 14 (at the same parameter distribution)] would have resulted in type 5 (at α = 0.1).

At the group level, type 1 participants exhibited a significant influence of grippiness on the COF, resulting in type 2. This is applicable to all data and average data. The insignificance of the type 1 data (“n/a” in Table 3) at the individual level is therefore very much dependent on the small number of data per participant (14 holds) and is affected by a high level of group noise.

The classification type (1–5) correlated significantly with the climbing experience (in years) through a positive trend (*R*^2^ = 0.1353, i.e., 13.5% of the classification type were explained from the climbing experience; *p* = 0.0921, α = 0.1). The correlations of the classification type with RP or OS were non-significant.

## Discussion

The main outcome of our research on climbing hold surfaces was that climbers judge the surface of a climbing hold from the perception of the grippiness rather than from the roughness profile. When designing the study, the authors heard different comments from climbers; some claimed that the roughness is the most dominant factor for assessing the surface of a hold, whereas others suggested the opposite. After climbing and after ranking, only 3 of the 22 participants indicated that the roughness is more important to them than the grippiness. From a perception point of view, the roughness profile can be easily felt simply from sliding the hand or the fingers over the surface. Conversely, the grippiness can also be felt easily by applying a slightly higher pressure and assessing the sliding resistance of the surface.

The individual perception of the holds’ surfaces was very diverse, with 50% of the climbers (type 1) lacking any correlation between the COF on the hold and the ranking of the surfaces (grippiness and roughness). The reason for this is unclear: whether the inability to subjectively rank the surfaces (misunderstanding of concepts) or the implicit inability of assessing the properties of the surfaces is responsible for the low correlation. Yet, at the group level, type 1 participants behaved like type 2 by exhibiting at least a significant correlation between grippiness and the COF. The only striking yet seemingly unsurprising result was that the single climber representing type 5 was a route setter. Route setters, in addition to having a vast experience in outdoor climbing on different rock faces, are dealing with a wide variety of indoor climbing holds for designing routes of varying difficulty. It is therefore expected that they are also more experienced in judging the surface of a hold and have a better understanding of surface properties.

By comparing the results of our study to the results obtained from other surface types, on the one hand, earlier studies confirmed that there is a correlation between the COF and the perceived roughness (Ekman et al., 1965) or the perceived slipperiness (Smith and Scott, 1996). On the other hand, recent studies on fabric texture perception mostly suggest that the apparent correlation seen is due to chance. For the following three recent studies, however, we had to further analyze the literature data to verify or reject a correlation.

Ramalho et al. (2013) measured the kinetic COF of five fabrics (polyamide, polyester, silk, cotton, wool) against the skin at loads between 0 and 1 N and a sliding speed 35 ± 10 mm/s. The participants of this study had to rank the fabrics with respect to four properties, among which were “rough/smooth” and “adhesive/slippery,” i.e., the same properties that were investigated in our climbing hold study. Ramalho et al. (2013) stated that “a positive correlation was obtained, especially concerning the slippery and the smoothness properties.” As the authors did not provide any data or statistics to support their claim, the data were extracted from their graphs (figures 4, 5 of Ramalho et al., 2013). Correlating both smooth and slippery rankings to the COF (multiple regression) resulted in a high *R*^2^ (0.7406); however, the regression was not significant (*p* = 0.2618), such that a correlation cannot be claimed to be established. The same result applied to the correlation of smooth ranking to the COF (*R*^2^: 0.5412; *p* = 0.1561). Only the slippery ranking showed a significant correlation with the COF (*R*^2^: 0.7401; *p* = 0.0616, α = 0.1).

Ding et al. (2018) investigated the roughness ranking of five fabrics (among other parameters) and their COF (measured at different speeds and loads with a steel ball probe). Roughness rankings and COF data were extracted from figures 2, 4 of Ding et al. (2018) and subsequently resulted in an insignificant correlation (*R*^2^: 0.0225; *p* = 0.8085).

Only the data of Chen et al. (2015) showed a significant correlation after further data analysis. The authors investigated the roughness ranking of 10 fabrics (among other parameters) and their COF (measured at 10 mm/s and 1.5 N with a commercially available “artificial finger”). As the data were not correlated, the roughness data were taken from Table 2 of Chen et al. (2015), and the COF data were extracted from Figure 7 of Chen et al. (2015). Correlating the data delivered a significant and positive correlation (the rougher, the higher the COF; *R*^2^: 0.7257; *p* = 0.0018).

The major difference between fabric perception studies and our climbing hold study is that for fabrics, the kinetic (sliding) COF is crucial (as fabrics slide along the skin), whereas climbing hinges on the “substatic” COF. Furthermore, the forces between skin and fabric are considerably smaller by several orders of magnitude compared to climbing. Finally, statistical evidence of correlations between subjective and objective parameters does not seem to be a priority in fabric perception.

To decide where this (statistical) inability comes from, the 14 different surfaces could have been investigated objectively as to grippiness and roughness (e.g., for comparing the objective results to the subjective ranking). This was not done for various reasons:

– The study deals with the climbers’ perception in the first place. What influences climbing is how the climbers subjectively perceive the surfaces’ properties and not how rough or grippy they objectively are.
– The grippiness or slip resistance can be assessed by determining the static COF at the point of impending slippage, which is pointless for various reasons. The static COF is load-dependent (force weakening and strengthening; Fuss, 2012). Applying the same average load (normal force) the climbers produced during climbing to the surface {i.e., average maximal force of 248 N [37.5% of the bodyweight (BW) on average] or average force of 150 N [22.8% BW]} and sliding the fingers or the palm over the surfaces up to the point of impending slippage would severely injure the skin on very rough surfaces (e.g., sandpaper). Furthermore, the kinetic COF does not necessarily decrease after the point of impending slippage but can increase such that climbers obtain an even better grip when sliding off a surface. This effect is known as velocity strengthening of the COF at lower sliding speeds followed by velocity weakening at higher sliding speeds (Fuss, 2012). Velocity strengthening was seen in surfaces of artificial climbing holds (Fuss and Niegl, 2012), as well as in the Teflon surface used in the present study (unpublished results). It is noteworthy to mention that these phenomena were found in fabrics as well (Ding et al., 2018).
– The roughness profile of a surface can be measured objectively but would be irrelevant if the COF cannot be determined objectively (owing to the reasons pointed out above), for comparative purposes.

Both grippiness and roughness influence the COF. Even if grippiness is directly related to the static COF, this does not explain the better correlation of grippiness and the COF found in this study. There are several reasons for this principle:

(1) The roughness of a surface profile also influences the COF as seen in the rough structures of antislip floor and tool surfaces and shoe sole profiles. In fact, Fuss and Troynikov (2012) found a significant positive power-law correlation between the *Ra* (arithmetic mean roughness) of pimpled rugby ball surfaces and their kinetic COF.
(2) Climbers apply to the hold a “substatic” COF rather than aiming for the static COF as known from Fuss and Niegl (2008a): the better the performance, the closer the climbers approach the static COF.
(3) The friction force is accentuated by the interlocking of a soft surface with a rough and harder surface. In climbing, this is achieved from the interaction of the fingers’ skin and the hold’s surface roughness for improving a firm grip. The formation of finger folds even improves the interlocking further.

As such, a “safe” grip on a flat and inclined surface can be judged by either, or both, surface property, i.e., grippiness and roughness.

The climbers reacted to the surfaces in different ways during or after climbing. Nine participants (40.9%) had no problems and succeeded with climbing all surfaces. Most of them did not make any comments. Climbers who slipped off one or more surfaces made comments related to the difficulty of getting a firm grip. It appeared that most of the climbers were surprised by the black rubber surface when climbing because of the high slip resistance despite the lack of roughness. Some climbers considered the surface variety as a new, different, and interesting way of experiencing climbing. Some stated that the reason they slipped is triggered by the thought that they could not hold on to a surface, resulting in eventual failure. A wider range of different surfaces is therefore also important for mental training of climbing.

To the best of the authors’ knowledge based on an extensive literature search (including review papers such as Orth et al., 2016, 2017), it seems that the research presented in this paper has never been done before. The applicability of our research results, however, has important implications. Artificial climbing holds and their properties were designed to mimic rock surfaces and structures (sandstone in most cases as they are made mostly from sand and resin with comparable surface roughness and porosity), thereby bringing the natural training facilities to the gym for reasons of accessibility. Why not approach the problem the other way around, namely by introducing surfaces and structures into the gym that are not common to rockfaces? This would enhance the training experience considering that gyms are predominantly created to facilitate training. Such an enhanced training process would be more versatile, holistic, and better suited for preparing the climber to quickly respond to extreme surface properties. Although it was already stated that more experienced climbers get closer to, but do not exceed, the static COP (as “experts are better than non-experts in picking up perceptual cues, as revealed by measures of response accuracy and response time”; Mann et al., 2007), in more general terms, “research has shown that perceptual-cognitive skills form an integral component of elite performance” (Klostermann and Mann, 2019). As such, perceptual training should be introduced to sport climbing, starting with surface perception.

## Conclusion

Perception is inherently a research area of psychology, specifically when it comes to conscious or implicit perceptions and how they are related to each other. We investigated the perception (both implicit and conscious) of the surface properties of climbing holds and identified that the perceived grippiness outweighs the perceived roughness in producing the amount of the COF applied to a surface. The grippiness is therefore implicitly more important than the roughness property.

The correlation between roughness and the COF was insignificant, whereas the correlation between grippiness and COF was significant at the group level. At the individual level, 50% of the participants did not show any correlations between surface properties and the COF; 36.4% exhibited correlations between the combined grippiness and roughness (multiple regression) and the COF, as well as grippiness and the COF; only 4.5% of the 36.4% showed an additional correlation between roughness and the COF. The results are interpreted in a way that climbers assess a hold’s surface based on the grippiness and not on the roughness, and apply a COF to the hold that reflects the grippiness perception.

## Data Availability Statement

The raw data supporting the conclusions of this manuscript will be made available by the authors to any qualified researcher, if they have obtained Ethics Approval for secondary use of existing data through a Consent Waiver.

## Ethics Statement

The studies involving human participants were reviewed and approved by the Swinburne University Human Ethics Committee (approval no. 20191290-1680) and adhered to the Declaration of Helsinki (Swinburne University of Technology, Melbourne, VIC, Australia). The participants provided their written informed consent to participate in this study.

## Author Contributions

All authors listed have made a substantial, direct and intellectual contribution to the work, and approved it for publication.

## Conflict of Interest

GN is employed by company Sportstättenverein Marswiese. The remaining authors declare that the research was conducted in the absence of any commercial or financial relationships that could be construed as a potential conflict of interest.
